# Notes and Comments

**Published:** 1884-08

**Authors:** 


### Notes and Comments.

Leprosy as seen in Brazil.—Dr. J. McF. Gaston, of Atlanta, in an interesting paper in the *New Orleans Medical and Surgical Journal* for May, on this subject, says:

My individual study of the disease has been confined to the province of St. Paulo, lying between 20° and 25° south latitude, in the Empire of Brazil, and having an opportunity to note the progress of it in three distinct localities at considerable distances from each other, during my stay at those several locations, it was not observed that there was any marked variation in its development from sectional modifications upon the subjects of leprosy.

The fact of the non-appearance of leprosy upon the lower lands of the province of St. Paulo does not quadrate with the reports of its appearance in Louisiana upon similar located regions, and we must, therefore, look to something else than altitude as an explanation of the growing prevalence upon the higher regions. What this depends upon cannot be satisfactorily determined, and the only plausible solution which presents itself is the greater preponderance of animal food, principally fresh pork, with lard as a large element of tempering in all their food, amongst the inhabitants of the table lands, where this disease prevails; whereas, in the lower territory fresh fish, with more vegetable food and fruits are used by the people who are exempt from it..

With these general remarks touching the conditions under which leprosy makes its appearance in the region which has been under my observation. I will proceed to delineate the features which mark its incipiency, progress and full development, with some of the measures that have been adopted in different stages of the disease.

The most constant feature in the outset of leprosy among the Brazilian population is greater or less local insensibility of the cutaneous surface upon circumscribed portions of the lower or upper extremities, and for the most part upon the fore-arm or leg. This is accompanied very soon by a want of the normal complexion of the part, assuming in the Caucasian race, a whiter aspect than the surrounding tissues, and in the Ethiopian race a dull ashy hue

Subsequently, or at times concomitantly, the color of the hands in the white subject is found, when in a dependent position, to be increased, so as to partake of a more florid hue at first, then a purplish tinge, and finally turning to a bronze complexion ; with the disappearance of this engorgement of the capillaries upon elevating the hand above the head. It is proper to note these abnormalties in the peripheral distribution of nerves and the capillaries in the incipiency of the disorder, as calculated to throw some light upon the nature of the disorder, which must be considered as a subject for further investigation by pathologists.

When these phenomena have existed for a few months, there will appear a thickening and hardening of the tissues in the lobes, or pendulous lower portion of the ears, the alæ of the nose, and about the eyebrows, with falling of the hair of the brow, that in the end become entirely depilated. As yet there has not occurred any marked discoloration of these tissues; but nodules are soon developed about the cheek bones and the forehead, with a slight coffee color in all the salient parts.

The hands and feet exhibit irregularities, partaking of the same hue, and there is some enlargement of the terminal appendages for a time, which soon, however, gives way to a diminution of their size from the shrinkage of the tissues; and there is a characteristic depression between the metacarpal bones of the thumb and forefinger, which ordinarily presents in the normal condition a protuberance, upon approximating the thumb with the forefinger, side by side, lengthwise.

At this stage there is usually a very considerable brownish discoloration of the ankle and lower portion of the leg, and yet strange to say the other portions of the body that are entirely protected by clothing, preserve their natural appearance even in the further progress of the degeneration of tissue about the face and extremities.

If depurative remedies are assiduously used, the disease may continue without any material aggravation for a year or two, with the appearances described, and in the meantime the patient is flattered with the delusive hope of an arrest of its development; but leprosy once set up in the system never retrogrades, and ere long, in spite of all the means used, it proceeds to disintegration.

The nodules and irregularities in different parts take on gradually a destructive ulceration upon the most prominent surfaces, and the degeneration extending to the deeper seated tissues, open running sores appear that are very offensive, not only to the sense of sight, but also to the olfactories.

In connection with this breaking down of the nodules, the extremities of the fingers and toes assume a gangrenous state, and the first phalanx of one or another falls off, and thus progresses in some cases until it reaches the articulation with the metacarpal and metatarsal bones, when death finally ensues.

There have been cases submitted to the constant and continued use of medication with various articles, such as lime juice, sarsaparilla, podophylin, iodide of potash, bichloride of mercury, biniodide of mercury, Donovan's solution, Fowler's solution, and other arsenical preparations, but without any decided or permanent effect.

Viewing the disorder as allied to the class of cutaneous diseases, the article that gave most promise of a good result was arsenic, and I have pushed it so far as to give fifty drops of Fowler's solution three or four times a day, with some apparent advantage for a time, yet the tolerance of the stomach for such doses ceased after its continuance for some weeks; and I have never been able to carry on the treatment so as to get the patient habituated to the article, as the population of the Alps become in using it after a considerable period of time.

Hygienic precautions, such as the regulation of the mode of living, with due regard to the articles of food, may avail as prophylactics in some cases, and thorough comprehension of the predisposing condition in the surroundings of those affected is demanded for any progress in the measures for averting this disease. But the closest observation and most mature study of its etiology has not thus far led to the adoption of any general plan that proves efficient in arresting the development of it when the germ is once implanted in the system.

In view of the possible extension of leprosy from persons affected with it, the exclusion of all suspected subjects of the disease becomes an imperative duty of the authorities of the United States, and a health officer competent to recognize the incipiency of this terrible disorder should be at each port of entry from those countries that are known to have it among their population. It would be the dictate of prudence to go still further and remove beyond the limits of our territory every case that may be discovered in any part of their country; and although an effort has already been made to provide for those unfortunates outside of the more populated regions of North America, the proximity of such settlements to the inhabitants still admits a more or less intercourse. The hygienic precautions should be carried to the extreme point of seclusion with all cases of leprosy; and though it may not be considered as coming directly under the category of a contagious disease, it most assuredly has an endemic quality that endangers those who reside in the immediate proximity of those affected with it, and the tendency to extension in certain sections may depend on a contamination of the surrounding atmosphere through the emanations from the bodies of those suffering with the disease by infection. This evil should be nipped in the bud, and the most vigorous measures should be adopted to stay its propagation throughout our land. Let Boards of Health look out this disease, with its hideous incurable development, and insist upon a vigorous preventive policy throughout the United States.

---

POISONING BY QUININE IN TYPHOID FEVER.—Dr. D. L. Lamb, of the Army Medical Museum, Washington, reports the following case in the *New York Medical Journal*, May 17th:

A little white boy, aged three years, had had slight diarrhœa and a little fever for about a week, but, as he kept about the house, his mother did not suspect serious disease, and therefore did not seek medical advice. About noon, July 11, 1877, he found a package of eighteen three-grain sugar-coated quinine pills, and, so far as could be learned, swallowed them all. Shortly afterward he took some rice and milk. About half an hour after swallowing the pills he complained of pain in the epigastrium. An emetic of mustard, salt and water was given, and he vomited some food and *one* pill. He *died* at 1.10 o'clock, apparently of syncope, and but little more than an hour after the ingestion of the pills.

It was deemed advisable to inspect the body, and, accordingly, an examination was made by me twenty-three hours after death. The body was well nourished, the skin of a citron tint. The head and spinal canal were not opened. The heart was relaxed, the right cavities were empty, the left contained a little fluid blood. The lungs were congested posteriorly. The liver and pancreas were normal. The Malpighian bodies of the spleen were well marked. The stomach was congested and presented a number of red ecchymoses; it contained some undigested rice, a thick, whitish liquid, and *one* pill, which was white and partly softened. The duodenum was much congested and contained milk coagula, a few grains of rice and *one* pill; Brunner's glands were conspicuous and white; the villi were hypertrophied. The upper part of the jejunum was much congested and contained milk coagula and *one* pill; all the solitary bodies and Peyer's patches were much thickened, and the peritoneal surface opposite the enlarged patches presented arborescent congestion. In the ileum the villi were hypertrophied, the solitary bodies and Peyer's patches progressively enlarged, and there was the same arborescent congestion as above mentioned; the contents were dark and granular. The cæcum and the ascending and transverse colon were congested, and the solitary bodies enlarged; the contents were soft and greenish, with some fruit-skins in the cæcum. There was a slight extravasation of blood in the gastro-colic omentum.

From the point of view of the intestinal lesions, the case does not admit of a doubt as to its being one of typhoid fever, although the clinical history is meager enough. The absence of medical attendance is unfortunate in depriving us of more exact data.

Four quinine pills of the eighteen are accounted for, one being vomited and three found at the post-mortem examination, leaving fourteen pills, or forty-two grains, which presented themselves for local effect and absorption to the stomach and the upper part of the small intestine. This portion of the alimentary tract responded in the form of congestion and ecchymoses. The coincidence of the relaxed heart and, apparently, death from syncope, point, as I understand, to the usual manner of death in poisoning by quinine.

---

A VERDICT OF MANSLAUGHTER AGAINST AN IRREGULAR PRACTITIONER.—The trial of "Dr." Franklin Pierce, an irregular medical practitioner of Worcester, Mass., occurred before the Superior Court in that city two weeks ago, on an indictment charging him with manslaughter in causing the death of Mrs. Mary A. Bemis, of West Boylston, in January, 1883, by the use of kerosene oil. The case excited considerable interest. The husband of the deceased testified that during his wife's illness Dr. Pierce said at various times that she had ulceration of the womb, gastro-bilious fever, typhoid fever, water about the heart, and erysipelas. He ordered that the patient's temperature be taken every hour with an ordinary house thermometer, and that a record of her pulse be also kept, so that he could report progress of the case to a dean of some college in Boston.

When he discovered the symptoms of erysipelas, one week before she died, he ordered the patient packed in kerosene oil from her neck to her toes, so as to draw out the disease. Cloths were saturated in oil, wrung out, and then laid on. She complained of intense burning pain, and the treatment was discontinued after two and a half hours. Dr. Pierce was sent for. He ordered the kerosene treatment renewed, this time by pouring oil on the cloths without removing them. This treatment was continued for thirty hours or more, when he ordered the cloths removed. It took three hours to remove the cloths, and large patches of skin came off with them. The next day Dr. Pierce presented a bill for $180 for consultation fees in Boston and $45 for surgical examinations. His services were dispensed with the following day, and other physicians called in, who said the patient was past recovery.

The testimony of the nurse showed, in addition, that after the first application of the kerosene the skin hung in strings from the patient's body. When the doctor ordered a second application he forbade the nurse to use sweet oil to allay the pain.

Dr. J. Marcus Rice, the medical examiner who conducted the autopsy, said the woman's epidermis was two-thirds burned away; that the true skin was destroyed in several places, and that in some spots the muscles were exposed. He found no sign of pericardial effusion. In his judgment death was caused by the destruction of the epidermis by the use of kerosene. This opinion was confirmed by other medical testimony. One physician who had treated Mrs. Bemis testified that pulmonary troubles and chronic diarrhœa would probably have ended her life soon, and kerosene packing only hastened death In his opinion she might have recovered from the kerosene treatment but for her vitiated condition.

The defense opened, and put on half a dozen witnesses, who, under Dr. Pierce's direction, have used kerosene in a similar manner to that in this case for erysipelas, typhoid fever, congestion, typhoid pneumonia, and other diseases. All agreed that the treatment was highly beneficial to them. The defense also offered testimony to show that the husband had administered two doses of aconite to his wife, early in her sickness, of eight drops each.

The only witness in rebuttal testified that, under the prescription of the defendant, kerosene oil had been used on her son as a remedy for lung fever until it took off the flesh and laid the bone bare.

The jury were out ten minutes and returned a verdict of guilty. —*Boston Medical and Surgical Journal.*

From the address on Ophthalmology presented to the American Medical Association May, 1884, by Julian J. Chisolm, M.D., of Baltimore, we extract the following sound words of advice to general practitioners:

What the general practitioner most needs is not a detailed account of scientific researches, nor even the narration of the novelties in practice which the last year has developed. What is of the most value is a familiarity with well established facts of practical use for the recognition and cure of serious diseases. How to use remedies aright is of the greatest importance. Take atropia, for instance, which is invaluable for the successful treatment of some of the most destructive inflammations of the eye, but it is far from being the universal panacea for every eye affection, as some professional gentlemen seem to think.

What I have said for the eye, I may equally duplicate for the diagnosis of ear diseases Many of these to be sure are very obscure, and can with difficulty be reached even by the process of exclusion. .Yet, hid away as this organ is in its labyrinthine portions, it can be as successfully studied as any of the other hidden organs of the body. We have, however. a portion of the ear under direct ocular exploration. The external ear, so-called, and the drum-head at the bottom of it, can certainly be as thoroughly studied as the skin in the palm of the hand. Everybody has heard of the drum, and every physician knows where the drum-head is to be looked for, but very few have ever seen it, to know its healthy from its diseased appearances. Many cannot recognize the drum-head at sight, or know whether they are looking at the skin lining the ear passage, or the drum-head at its extremity. They do not remember, when the knowledge is most needed, that the healthy drum membrane always exhibits a bright spot of reflected light at the lower end of the long process of the maleus, and that the sight of this marks with certainty its exposure upon inspection. Were this fact better known, ceruminous deposits concealing the membrane and causing deafness, would not be sent to specialists by good physicians, so that the former may secure great reputation in restoring hearing through means of a syringe and warm water. I have had patients travel from their homes and back again, from Texas to Baltimore, fully 3,000 miles, to go through this operation, because the family physician, a gentleman of very large experience and one well-read in his profession, was not sure of the appearances of a drum membrane, although thoroughly familiar with the book anatomy of the hearing organ. Make it a rule, without a single exception, to examine the external ear of every patient who complains of deafness, and not send them to a specialist until you are convinced that the disease lies beyond the drum-head. The general practitioner should be as skillful as the specialist to detect ceruminous deposits and to remove them.

I would urge upon the profession at large to appropriate to their own uses the simple truths which special medicine has made so easily understood. Make up your minds that as to the common diseases which attack the eye and the ear you will become as familiar with them as can be any specialist, and that hereafter no mistake shall be made in the different diagnosis of conjunctivitis and iritis as long as a solution of atropia can be obtained. Be firm in the determination that if any credit or reputation is to be gained by the restoring of hearing through the removal of excretions in the external auditory passage, no specialist shall rob you, at least, of this award. In other words, make the peculiar knowledge of the specialist the common knowledge of the profession.

---

A CASE OF ANURIA PERSISTING FOR FIFTEEN DAYS WITHOUT INJURIOUS EFFECTS.—Dr. L. Chappot de la Chanome, in *Le Concours Medical*, cites a case occurring in a man aged 66 years, who complained of pains in the lumbar region, general discomfort, and loss of appetite. There was no fever, and the tongue was natural. He prescribed a saline purgative, and friction over the painful region. The next day the patient declared that he had not passed urine for three days, but that it did not trouble him, as he felt no desire to do so. Palpation showed that the bladder was empty, or nearly so; but to make sure, he introduced a catheter, without producing a single drop of urine. He prescribed energetic diuretics, but without result. Six days later he called Dr. Bonin, of Monchamp, in consultation, and catheterism was again practiced, without effect. The prognosis naturally was very grave. The patient kept to his bed, had no fever, but a strong dislike to food and a decided relative weakness. He talked freely with those around him, and complained somewhat of lumbar pains. Diuretics and sudorifics were freely used, strychnia was given, the lumbar region was blistered, and warm baths were administered. Every day there was a moderate amount of perspiration, of an odor like that which is found in some cases of rheumatism, and each day there were two or three stools of the character of an ordinary diarrhœa. Finally, on the fifteenth day of the anuria the patient felt for the first time a pressing desire to urinate, and he hardly emptied his bladder before the desire returned, and the urinal was filled more than once. This continued for the first twenty-four hours. The second and third days found the amount diminished, but much more considerable than under ordinary circumstances. After that the secretion became normal, the health of the patient was rapidly re established, and the patient has been perfectly well ever since (eighteen months).—*Journal of the American Medical Association.*

---

M. PASTEUR.—At this time there is not a more conspicuous character in the scientific world than M. Pasteur, the distinguished Frenchman, whose numerous experiments and investigations have awakened such universal interest. It may not be uninteresting to our readers to learn something of the life of this well known individual. Louis Pasteur was born December 22, 1822. Early in life he began the study of chemistry and soon made important discoveries in this branch of science. He was made an assistant to the professor of chemistry at Strasburg, and subsequently at the age of thirty-two was made Dean of the Faculty of Sciences at Lisle. Whilst at Lisle he began the study of the subject of fermentation, and conducted the remarkable experiments which have opened up a new world to science in this field of research. His attention seems to have been drawn to this subject by the fact that the manufacture of alcohol was one of the chief industries in this department.

Later on he began to study the subjects of vaccination, of spontaneous generation, of wine, of the diseases of the silk-worm, of beer, chicken cholera, malignant pustule, septicæmia, and quite recently of rabies. The vast light which he threw on these subjects, and the important and valuable facts obtained by his investigations, are well known.

His doctrine of an attenuated virus of certain malignant diseases of inferior animals has created a new revelation in medicine and led to most important and valuable discoveries which are being made manifest in other departments of scientific research.

In 1868, M Pasteur brought on hemiplegia from overwork, from which he has never entirely recovered, the members of his right side being still impaired in motion. He has not discontinued his labors in consequence of this affliction, but, as is well known, has made some of his most important discoveries since 1868. In the conduct of his investigations he has been liberally aided by the French Government The ancient garden of the old college Rollin was placed at his disposal, and there he has stables for sick horses and diseased sheep, kennels for dogs with hydrophobia, and an abundance of hens, chickens, rabbits and guinea pigs for conducting his different experiments. It has been by the liberal use of animals that M. Pasteur has been enabled to arrive at the valuable facts he has from time to time made known to the scientific world. That these facts have an incalculable value to science no one can deny. His experiments with charbon alone have been the means of saving millions of dollars annually to the wool growers of France.

M. Pasteur's services have been liberally rewarded by pensions from the French Government, and the scientific world has accorded to him the highest distinctions within its gift.

As might be expected, M. Pasteur has aroused the enmity and antagonism of the anti vaccinationists and those fanatics who deny the right of science to experiment with animals. Necessarily M. Pasteur has sacrificed the lives of many dumb brutes and inflicted in this way much suffering, yet we have his own word for it that in all vivisections the animals were chloroformed. He says: 'Never have I had the courage to kill a bird in hunting; but when experiments are concerned no scruple stops me. Science has the right to invoke the supremacy of the end." Such language is well worthy of consideration by those persons who object to vivisections and vaccinations on the ground of unnecessary cruelty to animals. Such in brief is the history of the man who has been characterized by the famous American citizen, Mr. Henry Bergh, as a "merciless empiric."—*Maryland Medical Journal.*

---

A MONSTER.—In Dalldorf, Germany, a monster was born with the following peculiarities: It was a boy, and lived fourteen hours; it had 16 fingers, eight on each hand, thumb and little finger being double, but the two parts were grown together, and the other fingers were united by a web; otherwise nails and joints normal. It also had sixteen toes, crooked and with joints not fully developed, but nails and single toes plainly discernible. The face evinced a cleft palate, but the most remarkable fact was the utter want of a tongue, while two teeth each were present in front in the upper and in the lower jaw.—*Med. and Surg. Reporter.*

HOW TO TAKE A PILL.—Dr. Samuel E. Wills, Earlville, Md., suggests the following method: "Having noticed that if a person at meals inclined the head backwards, as in laughing, while there was food in the mouth, they were pretty certain to be strangled from 'the food going the wrong way,' I instructed those of my patients who had difficulty to swallow pills, to *keep* the head in the position they would if eating and swallowing food at the table—that is, the head inclined forward, the chin near the breast—and *keep* it in that position. If a small portion of saliva be on hand, or a small quantity of water taken after the pill is put in the mouth, it will surprise the patient and gratify the doctor to witness the facility with which it will be swallowed. To direct the patient to keep his eyes on his toes, I have found a help to keep the head in the proper position."—*Medical and Surgical Reporter.*

---

THE RESULT OF SWALLOWING NEEDLES.—The following report is taken from the *Wiener Med. Blat.* a very unique case, but is well authenticated:

Patient, domestic, aged 23; phthisical family history. In 1870 she suffered from rheumatism, for which she was treated, and when she was discharged there were symptoms of heart disease, but aside from this she was perfectly healthy. In the spring of 1882 she complained of a feeling of weakness, pain in the head and limbs, and symptoms of angina. During the summer gastro-intestinal catarrh set in, so that the patient was again in the hospital for four weeks. In the fall of the same year patient was again admitted, complaining of a stiffness in the back and lower extremities; she could not turn in bed without assistance. Soon very obstinate attacks of vomiting set in, independent of anything that was ingested, constipation, pain in the abdomen, and sensitiveness in the region of the cæcal valve. Later, there were attacks of tonic spasm with hemiparesis and hemianesthesia. The diagnosis of hysteria was made. In June, 1883, severe diarrhea and vomiting set in, with sharp stinging pain in the knee, left arm, and in many other parts of the body. The formation of an abscess below the knee caused her to be transferred to the surgical department of the hospital. The abscess was opened and an ordinary needle was extracted. In other parts of the body discolored painful spots were now found, in which a hard foreign substance could be felt. Patient was chloroformed, and from her arms, breast and abdomen twelve and a half needles were extracted. Patient would not say how the needles got there. she was very reticent, but later she confessed that in May, 1881, she wanted to commit suicide, therefore swallowed five and a half packages of needles, each package containing twenty-five needles. They were swallowed wrapped in paper with the thick end first. She required two weeks to swallow them all. For a long time after she felt nothing of the needles, then pain in the abdomen and bowels set in, confining her to bed for two months. She watched her stools carefully, and claims never to have passed any needles per rectum. At that time she absolutely refused all medical aid for fear of being discovered. On nineteen different occasions, many times under chloroform, sixty-five whole and six half needles were extracted. In January, 1884, she was again operated on, removing forty-one needles, the whole number being one hundred and ten. This patient now became severely hysterical again, and when closely questioned as to the cause, she confessed that during the past Christmas she swallowed five more packages of needles, containing no less than twelve needles apiece.—*C. W. T., Lancet & Clinic.*

---

SACRACHE AND BACK-ACHE.—In the course of some interesting lectures by Dr. J. Mathews Duncan, in the *London Medical Times*, we note the following, which is possessed of practical interest:

There is no more common, and therefore no more important, symptom of uterine disease, and especially of disease of the neck of the womb, than sacrache. The pain is dull, or an ache rather than pain; it is situated at or near the base of the sacrum; and, referring to it, patient puts her hand to the part. I say with emphasis "at or near," and it is desirable to make this more definite. A pain below the middle of the sacrum is not at or near, and a pain above the middle of the lumbar spine is not "at or near." Such pains and aches do not point to the womb as the characteristic sacrache does; but the characteristic sacrache only points in that direction; it is not in itself nearly sufficient, not even strong, evidence of disease of the womb. Occurring in a virgin it would not, alone, unless very severe and inveterate, lead you to make an actual examination of the womb.

Other back-aches, that is, pains in other regions of the back, not the sacrache, may accompany uterine disease, but do not point to the womb, are not symptoms rationally held as indicating womb disease. Unfortunately women are, at present, so under the influence of bad medical instruction, that they regard all pains in the back, from the occiput to the coccyx, as nearly sure indications of uterine mischief, and demanding uterine treatment

The sacrache of womb disease may be constant, but generally it comes and goes. Frequently it is dispelled by lying, is felt on going to bed, and has disappeared before morning, and long standing makes it reach its highest pitch. When it is otherwise, that is when the ache is worse in the morning before getting out of bed, or is relieved by walking, then it is certainly not uterine.

I have said that you must not regard all sacraches or other backaches as uterine. They are common in men and in women. A weakly woman, who attends to all her pains, can do no standing or walking without back-ache, and often it is a sacrache; especially, if she has a long back, will she suffer in this way.

The pains liable to be confused with real sacrache are all in the lower back, about the lumbar spine; it is only such that might mislead any rational physician. Regarding them you will get some light from noticing the causes of the same pains in men. Now I find that weakly men are liable to these aches. sacral or lumbar, on walking or standing; and in many they are produced by excessive venereal indulgence.—*Medical and Surgical Reporter.*

---

TONGALINE —The value of tongaline in the treatment of neuralgia and rheumatism is established by many careful observers in this country as well as by Ringer and Murrell of London, England. The use of the preparations of opium in neuralgia, especially, is open to the great danger to the patient of the establishment of the "opium habit." An agent preventing this, and which is almost a specific in the acute forms of neuralgia, is to be welcomed and employed by every physician.—*Buffalo Medical and Surgical Journal.*

---

VACCINATION AS A PREVENTIVE OF HYDROPHOBIA AND YELLOW FEVER.—While M. Pasteur claims to have more than twenty dogs which he has rendered no longer susceptible to attacks of hydrophobia by inoculating them with the virus of that disease, after it had been attenuated or diluted by passing through several monkeys and rabbits; Dr. Domingo Freire, of Rio Janeiro, claims to have discovered the specific virus of yellow fever, and after inoculating some of the lower animals with it, he had used the diluted virus for vaccinating several hundred persons, and rendering them unsusceptible to the fever. Dr. Freire represents the specific virus or cause of yellow fever to be a vegetable parasite, which he has named cryptococcus xanthogenius. By what process his discovery was made we are not yet informed; neither have we sufficient knowledge of the tests to which the four hundred inoculated persons have been subjected, to establish our faith in the reliability of the claims, so confidently expressed.—*Journal of the American Medical Association.*

---

MELLIN'S FOOD.—We have had occasion to inspect a recent analysis of Mellin's Food by Professor Fresenius, of Weisbaden, and here give an abstract of his results.

The preparation is a moderately fine, yellowish-white, hygroscopic powder. It is not completely soluble in water, but is almost completely so—with the exception of a trace—in the stomach. The constituents are as follows:

| | | |
|---|---|---|
| I. Soluble in water: | | |
| Non nitrogenized, organic. | | |
| Maltose and Dextrose (33.46+35.92) | 69.38 | |
| Nitrogenized, organic. | | |
| Albumen (2.13), Peptone (0.87), Amides (1.69) | 4.69 | |
| Inorganic | 4.23 | |
| | | 78.30 |
| II. Insoluble in water, but almost completely dissolved in the stomach: | | |
| Non-nitrogenized, organic. | | |
| Fat (0.08), Cellulose, etc. (3.10) | 3.18 | |
| Nitrogenized, organic | 5.06 | |
| Inorganic | 0.14 | |
| | | 8.38 |
| III. Water, including loss by drying at 120° C | | 13.32 |
| | | 100.00 |

As a matter of analytical interest, it may be added that the albuminoids were determined by Prof. Fresenius in the following manner: The albumen is calculated from the nitrogen of those nitrogenized substances which are precipitable by cupric hydrate in a solution containing a slight excess of acetic acid. The calculation is made by multiplying the nitrogen with 6.25. The peptones are found in a similar manner by calculation from the nitrogen obtained from the precipitate produced by phosphomolybdate of sodium in the filtrate from the preceding operation, after acidulating with hydrochloric acid. The amides result from the difference of the sum of nitrogen of the protein-bodies, peptone and that obtained from the nitrogenized substances insoluble in water on the one hand, and the total nitrogen on the other hand Of the 9.75 per cent. of nitrogenized constituents, only 0.2 per cent. were found to be insoluble.—*American Druggist.*

---

GANGRENE OF THE PENIS.—The *Revue Medicale*, in its review of the affections of the genital organs, refers to a case of gangrene of the penis associated with circumcision, and a subsequent examination revealed the presence of glycosuria. The circumcision was performed on the 8th, gangrene set in on the 11th. Intense fever followed, delirium, phlegmonous erysipelas and death on the 28th. The lesson associated with this is evident.

One case of gangrene, reported by M. Leteinturier, is unique. A young man, believing in the magic influence of his mistress' ring, passed it over his penis, but found himself in a tight place, from which he could not extricate himself, and paid for his devotion by the gangrene of the integument of the penis and a part of the scrotum. Another individual, in order to avoid the risk of impregnating his mistress, securely tied the end of the prepuce in front of the gland. At the expiration of a coition under these circumstances inflammation set in, which resulted in gangrene.—*Medical Review.*

---

TRACTION SUTURE.—Dr. Allis, in the Annals of Anatomy and Surgery, says that when a large portion of integument has been cut away, as in removal of the female breast, the healthy borders sometimes can not be fully approximated; and even an attempt to do so is accompanied with such a degree of tension that the sutures soon cut their way out. To distribute this tension, after drying the skin thoroughly, he applies strips of adhesive plaster from the margin of the wound in the direction he wishes the sutures to hold. He then passes his needle deeply through plaster and skin. After the sutures are in position, and before tightening them, he requests an assistant to approximate the margins of the wound by pressure from his hands, while he secures them by twisting the wire.—*Louisville Medical News.*

THE Micrococcus of Yellow Fever has been discovered by Dr. Domingos Freire of Rio de Janeiro He claims to have discovered it in the vomit, the blood, and different secretions of yellow fever patients, and calls it micrococcus xanthogenius. This organism can be found in abundance in the earth of a cemetery at Rio Janeiro, and even in the soil of the whole city. The doctor has succeeded in inoculating several animals with yellow fever, and by cultivating the virus to the fifth or sixth generation has attenuated it so that it becomes a vaccine. He has inspired enough confidence to vaccinate 400 persons with this product of successive cultures. This course has been severely criticised by the local physicians who do not believe in the innocuousness of these measures. It seems that this incident is causing quite a flutter, and that this bold experimenter is meeting with a number of partisans who uphold his views.—*Medical Review.*

---

RECOVERY AFTER TRANSFIXION OF THE HEART.—An elderly lady with suicidal proclivities for which she was under treatment in a hospital, was found one evening, just after getting into bed, to have become suddenly and violently ill, and medical aid at once was summoned. On its arrival she was found to be quite unconscious, very pale, skin cold and clammy, pupils widely dilated, but there was no conjugate deviation present; the features were drawn and altered, and the head was drawn rhythmically from side to side, patient moaning at times The radial pulse on the right side was very weak, that on the left almost imperceptible; rate seventy-eight per minute. The left arm and leg were quite paralyzed, so far as could be made out. The right arm was feebly and aimlessly lifted and let fall now and then. She had vomited a little, but no deleterious substance could be detected in the ejected matter by the rough examination possible at the time.

On palpation over the precordia the cause of this rather puzzling condition was discovered. A knob about the size of a large pea was felt adhering to the chest-wall in the region of the apex beat, and this proved to be the head of a large steel shawl-pin, three and a half inches long, which the patient had succeeded in secreting, unobserved by the attendant in charge, and which she had thrust right into the chest at the situation mentioned above, the part imbedded being directed slightly upwards and inwards and measuring about two and three-quarter inches. It was at once removed and stimulants administered, and almost immediately the heart's action began to gain in force. The pulse became stronger and steadier, and consciousness slowly and steadily returned. For some time there was very urgent dyspnea, and she complained of much pain in the precordia, but these unfavorable symptoms diminished as time went on, and after an hour had passed she was much better, the paralysis of the left side had disappeared, the pulse was steady and of fair strength—rate one hundred and eight per minute—and she was quite conscious of all that occurred around her. After four hours she was sick again, and vomited a little, but this soon passed off and she fell asleep, and slept at intervals during the rest of the night, stimulants being frequently given in small doses. On the following day she complained of some pain at the site of puncture, and of headache, but was otherwise pretty well, and her recovery proceeded after this without an interruption. Some months have now elapsed. and no trace of the injury can be detected.—*Edinburg Medical Journal*, *Lancet and Clinic.*

---

A WINK AS GOOD AS A NOD.—Dr de la Pommerais was executed in June, 1864, for a murder of the Palmer type. On the night before his execution he was visited by Surgeon Velpeau, who, after a few preliminary remarks, informed him that he came in the interest of science, and he hoped for Dr. de la Pommerais' co-operation. "You know," he said, "that one of the most interesting questions of physiology is as to whether any ray of memory, reflection or real sensibility survives in the brain of a man after the fall of the head." At this point the condemned man looked somewhat startled; but professional instincts at once resumed their sway, and the two physicians calmly discussed and arranged the details of an experiment for the next morning. "When the knife falls," said Velpeau, "I shall be standing at your side, and your head will at once pass from the executioner's hands into mine. I will then cry distinctly into your ear, 'Count de la Pommerais, can you at this moment thrice lower the lid of your right eye, while the left remains open?'" The next day, when the great surgeon reached the condemned cell, he found the condemned man practicing the sign agreed upon. A few minutes later the guillotine had done its work, the head was in Velpeau's hands and the question put. Familiar as he was with the most shocking and ghastly scenes, he was almost frozen with terror as he saw the right lid fall, while the other eye looked fixedly at him. "Again!" he cried frantically. The lids moved but they did not part. It was all over.—*Med. Review.*

